# The effects of basal insulin peglispro vs. insulin glargine on lipoprotein particles by NMR and liver fat content by MRI in patients with diabetes

**DOI:** 10.1186/s12933-017-0555-1

**Published:** 2017-06-06

**Authors:** Trevor J. Orchard, Bertrand Cariou, Margery A. Connelly, James D. Otvos, Shuyu Zhang, Caryl J. Antalis, Tibor Ivanyi, Byron J. Hoogwerf

**Affiliations:** 1 Department of Epidemiology, GSPH, University of Pittsburgh, Pittsburgh, PA USA; 2 l’Institut du Thorax, CHU Nantes INSERM, CNRS, UNIV Nantes, Nantes, France; 3 LipoScience, Laboratory Corporation of America Holdings, Morrisville, NC 27560 USA; 4 Eli Lilly and Company, Lilly Corporate Center, Indianapolis, IN 46285 USA; 5 Eli Lilly and Company, Budapest, 1075 Hungary

**Keywords:** Diabetes, Basal insulin peglispro, Insulin glargine, Lipoproteins, NMR, Apolipoproteins, Liver fat, MRI, Adiponectin

## Abstract

**Background:**

In Phase 2/3 studies of basal insulin peglispro (BIL) compared to insulin glargine, patients with type 1 or type 2 diabetes previously treated with insulin and randomized to BIL had an increase in serum triglycerides (TGs). To further understand lipoprotein changes, a lipid substudy which included liver fat content was designed to assess relationships among the measured variables for each diabetes cohort and compare the hepato-preferential insulin BIL to glargine.

**Methods:**

In three cohorts of patients with diabetes (type 1, type 2 insulin naïve, and type 2 previously on insulin; n = 652), liver fat content (LFC) was determined by magnetic resonance imaging (MRI) and blood lipids were analyzed by nuclear magnetic resonance (NMR) spectroscopy at baseline, 26 and 52 weeks of treatment. Apolipoproteins, adiponectin, and other lipid parameters were also measured. Descriptive statistics were done, as well as correlation analyses to look for relationships among LFC and lipoproteins or other lipid measures.

**Results:**

In patients with type 1 diabetes treated with BIL, but not glargine, small LDL and medium and large VLDL subclass concentrations increased from baseline. In patients with type 2 diabetes previously on insulin and treated with BIL, large VLDL concentration increased from baseline. In insulin naïve patients with type 2 diabetes treated with BIL, there were very few changes, while in those treated with glargine, small LDL and large VLDL decreased from baseline. Baseline LFC correlated significantly in one or more cohorts with baseline large VLDL, small LDL, VLDL size, and Apo C3. Changes in LFC by treatment showed generally weak correlations with lipoprotein changes, except for positive correlations with large VLDL and VLDL size. Adiponectin was higher in patients with type 1 diabetes compared to patients with type 2 diabetes, but decreased with treatment with both BIL and glargine.

**Conclusions:**

The lipoprotein changes were in line with the observed changes in serum TGs; i.e., the cohorts experiencing increased TGs and LFC with BIL treatment had decreased LDL size and increased VLDL size. These data and analyses add to the currently available information on the metabolic effects of insulins in a very carefully characterized cohort of patients with diabetes.

*Clinicaltrials.gov registration numbers and dates* NCT01481779 (2011), NCT01435616 (2011), NCT01454284 (2011), NCT01582451 (2012)

**Electronic supplementary material:**

The online version of this article (doi:10.1186/s12933-017-0555-1) contains supplementary material, which is available to authorized users.

## Background

In diabetes and insulin resistance, the dysregulation of lipid metabolism can be detected in altered levels of circulating lipids. The introduction of insulin therapy may improve lipid metabolism and circulating lipids, raising HDL-cholesterol and lowering triglycerides (TGs) in patients with type 2 diabetes [1, 2]. Proton nuclear magnetic resonance (NMR) spectroscopy offers a more detailed look at changes in lipid metabolism by measuring individual lipoprotein subclasses. There are limited data on the effects of insulin on lipoprotein subclasses in type 1 or type 2 diabetes.

Basal insulin peglispro (BIL) is a novel long-acting insulin analog with a large hydrodynamic size [3] and hepato-preferential action due to reduced peripheral effect [4, 5]. This reduced peripheral effect on glucose disposal suggests a reduced effect on lipogenesis; increased lipolysis in insulin-treated patients who were changed to BIL likely contributed to the observed lipid changes. In patients previously on basal insulin and randomized to BIL in Phase 2 studies, an increase from baseline in serum TGs was noted [6, 7]. In the Phase 3 program, serial lipid profiles were obtained to further characterize and understand the time course for the potential changes in lipid metabolism in response to BIL [8]. In the overall BIL program at baseline, patients with type 2 diabetes on insulin had nominally lower TG values (mean range 144–149 mg/dL) than insulin naïve patients with type 2 diabetes (mean range 159–161 mg/dL); patients with type 1 diabetes had lower TG values (mean range 83–91 mg/dL) than patients with type 2 diabetes. At 26 weeks of treatment, patients with type 2 diabetes randomized to insulin glargine had reductions in mean TG (insulin naïve: −11 mg/dL; prior insulin use: <4 mg/dL); patients with type 1 diabetes had reductions of <2.0 mg/dL. At 26 weeks of follow-up, patients randomized to BIL had increases in TG (insulin naïve: 3.6 mg/dL; all patients previously treated with insulin and then randomized to BIL: 19‒24 mg/dL).

A subset of patients from four Phase 3 studies representing different patient cohorts were recruited to participate in a lipid substudy; these patients are the focus of this report. They had blood samples analyzed by NMR for lipoprotein particle subclass concentration and size determination [9] and underwent assessment of liver fat content (LFC) by MRI [10]. Additional parameters, such as plasma fasting free fatty acids (FFA), apolipoproteins, serum total cholesterol efflux capacity (CEC), cholesterol ester transfer protein (CETP) mass and activity, and plasma adiponectin, were also measured.

The primary LFC and routine blood lipid results are reported elsewhere [8, 11]. Here we report the results of the lipoprotein particle and related lipid analyses by cohort (type 1 diabetes, type 2 insulin naïve, or type 2 previously on insulin) and treatment (insulin glargine or BIL) at baseline and follow-up. We also examine the relationships among lipoprotein particles and LFC.

The primary objective of these substudies was to compare the treatment effects of BIL vs. glargine on the change from baseline in LDL particle concentration at 52 weeks. Secondary objectives included comparing the concentration and change from baseline of LDL particles, HDL particles, intermediate density lipoprotein (IDL) particles, and very low density lipoprotein (VLDL) particles, as well as the values, changes from baseline, and associations with liver fat content of the following parameters: serum total CEC, serum FFA, CETP activity and mass, apolipoproteins [Apo A1, Apo A2, Apo B100 (calculated using Apo B total and Apo B48), Apo C3], and total adiponectin. Changes in particle size of LDL, HDL, and VLDL were also compared. The objectives and results of the LFC study have been reported elsewhere [11, 12].

## Methods

### Patients and studies

The lipid/MRI substudy was conducted as protocol addenda to four Phase 3 clinical studies of BIL compared to glargine in patients with type 1 or type 2 diabetes [13–16]. Three patient cohorts were represented (type 1 diabetes [IMAGINE 1 and IMAGINE 3], type 2 diabetes previously taking insulin (and then randomized to either glargine or BIL, described as the “basal switch” cohort [IMAGINE 5]), and type 2 diabetes insulin naïve [IMAGINE 2]). All four studies were multinational and randomized; IMAGINE 2 and IMAGINE 3 were double-blind. Common features of the Phase 3 studies included intensive insulin adjustment with similar basal (and bolus in type 1 diabetes) insulin adjustment algorithms, patient inclusion and exclusion criteria, no changes in lipid-altering medications up to week 12, and stable doses of background glucose-lowering medications before and during the study. Exclusion criteria included fasting hypertriglyceridemia (defined as serum TGs >400 mg/dL). In addition, if a patient developed elevated fasting TG >600 mg/dL at any time during the trial, the patient was discontinued from the study treatment.

### Laboratory methods

For all of the analyses in the lipid substudy, blood was collected after an approximately ≥10-h fast. Lipoprotein particle concentrations and sizes were measured at Liposcience (now Laboratory Corporation of America Holdings, Morrisville, NC, USA) by *NMR LipoProfile* analysis using the LP2 algorithm as previously described [9]. The liver fat content was measured by MRI as previously described [11]. Global CEC was performed by Vascular Strategies LLC, Plymouth Meeting, PA, USA as previously described [17, 18]. CETP mass (ELISA) and activity (fluorometric assay) were determined by Pacific Biomarkers, Seattle, WA, USA. Apo A1, Apo B and Apo B48 were determined by nephelometry by Siemens Healthcare GmgH, Erlangen, Germany. Apo-B100 was calculated as the difference between Apo-B and Apo-B48. The following assays were performed by Covance, Princeton, NJ, USA: serum free fatty acids (enzymatic), Apo A2 and Apo C3 (turbidometric immunoassay), and adiponectin (Quantikine Human Adiponectin/Acrp30 Immunoassay, R&D Systems, Minneapolis, MN).

### Liver fat content (LFC) measurements

LFC was evaluated by MRI. For each subject, all scans were obtained using the same scanning techniques, equipment, and imaging parameters as at baseline. The entire liver was scanned with 3–5 overlapping series in the axial plane. LFC was estimated using six-echo images with spectral model and T2* correction, at sequential alternating out-of-phase and in-phase echo times, as previously described [10]. To ensure consistent interpretation of scans, phantoms containing liquid of varying fat fractions were used for quality control, and study images were centrally evaluated by a qualified vendor (Virtual Scopics Inc. Rochester, NY, USA).

### Statistical methods

Statistical software used was SAS version 9.1 or higher (SAS Institute Inc. Cary, NC, USA). Analyses were conducted on all randomized patients in the lipid substudy who took at least one dose of study insulin. A mixed-model repeated measures (MMRM) model was used to analyze continuous variables collected at multiple post-treatment time points with terms for treatment, baseline values of the analysis variables, stratification factors for randomization, week, and treatment by week interactions. Values are presented as least squares mean (LSM) ± standard error (SE) unless otherwise noted. All treatment differences are reported as LSM difference (BIL-glargine) with 95% confidence intervals (CI). For treatment comparison at baseline, an analysis of variance model was used for continuous variables and Fisher’s exact test for categorical outcomes. Spearman’s correlation analyses were performed to assess the relationships between NMR parameters and LFC. To adjust for multiplicity, statistical significance is defined as two-sided p value <0.001. Raw data was analyzed as it was collected without transformation or exclusion of outliers. Missing data was handled through MMRM analysis without explicit imputation.

## Results

### Patients

A total of 652 patients from 4 Phase 3 studies of BIL versus glargine comprised the full analysis set for the lipid substudy; 219 were from the glargine arms and 433 from the BIL arms. Baseline demographic and clinical characteristics, NMR lipoproteins, and other lipid parameters for the three patient cohorts are summarized in Table 1. Within each patient cohort there were no major differences between treatment groups at baseline. Six patients in the lipid substudy discontinued from study treatment because of a post-baseline fasting TG >600 mg/dL, per the protocol; all were in the BIL treatment group.

**Table 1:** Baseline demographic and clinical characteristics, lipoprotein subclasses, and other lipid parameters

Patient cohort	Type 1 diabetes		T2 insulin naive		T2 basal switch	
Treatment, n	Glargine	BIL	Glargine	BIL	Glargine	BIL
	n = 86	n = 163	n = 59	n = 119	n = 74	n = 151
Demographic and clinical data						
Age, years	38.6 ± 13.8	40.0 ± 12.5	57.6 ± 9.5	58.5 ± 10.1	62.4 ± 8.7	62.0 ± 8.5
Female, % of patients	38.4	44.8	39.0	33.6	45.9	44.4
White, %	90.7	87.7	83.1	79.0	98.6	93.4
Duration of diabetes, years	16.5 ± 10.9	18.5 ± 12.0	12.1 ± 7.7	11.1 ± 6.8	13.3 ± 6.9	12.8 ± 6.5
BMI, kg/m^2^	26.5 ± 4.3	26.3 ± 3.7	32.5 ± 5.4	33.3 ± 4.9	32.9 ± 5.1	31.8 ± 4.8
HbA1c, %	7.9 ± 1.2	7.9 ± 1.1	8.3 ± 1.0	8.5 ± 0.9	7.4 ± 0.8	7.4 ± 0.8
Triglycerides, mg/dL	93 ± 56	94 ± 93	165 ± 91	167 ± 91	152 ± 73	168 ± 141
LDL-C, mg/dL	106 ± 29	104 ± 29	93 ± 31	89 ± 34	99 ± 32	100 ± 40
HDL-C, mg/dL	59 ± 16	63 ± 16	46 ± 12	47 ± 13	46 ± 13	48 ± 12
Total-C, mg/dL	184 ± 37	186 ± 36	171 ± 38	169 ± 37	175 ± 36	180 ± 48
Statin use, % of patients	16.3	18.4	62.7	63.0	58.1	60.3
Liver fat content, %	3.32 ± 3.54	3.09 ± 3.13	12.7 ± 8.07	13.3 ± 8.75	9.96 ± 8.43	10.4 ± 7.54
Lipoprotein concentration						
HDL-P, μmol/L						
Total HDL	32.0 ± 4.91	33.2 ± 6.04	32.3 ± 5.48	32.9 ± 6.12	30.8 ± 5.64	33.0 ± 5.65
Large HDL	8.41 ± 3.57	9.55 ± 3.62	4.80 ± 2.69	5.21 ± 2.95	5.27 ± 2.84	5.45 ± 2.94
Medium HDL	3.84 ± 3.68	3.49 ± 3.67	2.31 ± 2.51	2.80 ± 3.10	2.67 ± 4.00	2.66 ± 3.33
Small HDL	19.8 ± 5.96	20.1 ± 6.42	25.2 ± 5.71	24.9 ± 5.25	22.9 ± 5.17	24.9 ± 5.41
LDL-P, nmol/L						
Total LDL	1084 ± 369	1030 ± 315	1213 ± 343	1209 ± 411	1231 ± 329	1227 ± 401
IDL	36.6 ± 41.2	30.7 ± 36.1	50.6 ± 41.7	46.1 ± 39.2	52.9 ± 41.6	52.6 ± 49.5
Large LDL	424 ± 188	471 ± 205	224 ± 159	243 ± 182	281 ± 180	295 ± 209
Small LDL	624 ± 391	528 ± 344	939 ± 369	919 ± 408	898 ± 348	880 ± 391
VLDL-P, nmol/L						
Total VLDL	54.4 ± 32.3	48.3 ± 34.9	75.6 ± 39.4	75.8 ± 39.4	77.8 ± 35.7	76.8 ± 38.7
Large VLDL	1.69 ± 3.01	1.69 ± 4.34	4.74 ± 5.11	4.81 ± 4.92	3.90 ± 3.98	4.44 ± 4.56
Medium VLDL	18.3 ± 16.3	15.1 ± 17.1	34.2 ± 26.7	34.4 ± 25.9	34.2 ± 22.1	32.0 ± 21.6
Small VLDL	34.4 ± 18.6	31.6 ± 21.8	36.6 ± 17.2	36.5 ± 17.6	39.8 ± 17.8	40.4 ± 21.8
Lipoprotein sizes						
HDL-P size, nm	9.14 ± 0.47	9.26 ± 0.49	8.62 ± 0.38	8.65 ± 0.32	8.72 ± 0.38	8.73 ± 0.38
LDL-P size, nm	21.2 ± 0.78	21.4 ± 0.76	20.3 ± 0.61	20.3 ± 0.76	20.5 ± 0.72	20.5 ± 0.73
VLDL-P size, nm	49.2 ± 8.39	49.9 ± 9.19	51.9 ± 8.32	52.9 ± 7.68	51.0 ± 7.64	51.2 ± 8.77
Apolipoproteins						
Apo A1, mg/dL	156 ± 26.5	165 ± 27.6	140 ± 22.7	142 ± 23.8	138 ± 24.7	146 ± 24.6
Apo A2, mg/dL	39.7 ± 6.48	40.0 ± 7.30	37.1 ± 6.33	37.4 ± 7.07	34.4 ± 5.39	37.1 ± 6.13
Apo B100, mg/dL	82.0 ± 20.4	81.1 ± 21.0	86.0 ± 21.0	85.8 ± 23.7	89.0 ± 21.1	89.2 ± 26.7
Apo C3, mg/dL	9.87 ± 3.54	10.1 ± 4.25	13.0 ± 5.33	12.7 ± 5.51	12.7 ± 4.41	12.8 ± 4.74
Other parameters						
Adiponectin, ng/mL	10,285 ± 6947	12,124 ± 7733	6690 ± 6315	5527 ± 5794	5950 ± 3903	6307 ± 4616
CETP, pmol/mL/min	22.1 ± 6.43	22.0 ± 5.77	18.41 ± 4.44	20.0 ± 6.10	19.2 ± 5.03	18.3 ± 5.70
CETP, μg/mL	2.36 ± 0.55	2.40 ± 0.62	1.86 ± 0.44	1.94 ± 0.58	2.13 ± 0.49	2.10 ± 0.53
Serum CEC, %	11.4 ± 3.49	12.1 ± 3.12	11.7 ± 2.88	11.4 ± 3.02	12.7 ± 3.47	12.9 ± 3.05
Free fatty acid, mEq/L	0.54 ± 0.35	0.56 ± 0.32	0.59 ± 0.18	0.64 ± 0.24	0.56 ± 0.23	0.59 ± 0.22

Within each cohort, there were no significant differences between treatment groups in baseline data. Data are mean ± SD unless otherwise stated

*CEC* cholesterol efflux capacity, *CETP* cholesterol ester transfer protein, *LDL-C* low density lipoprotein cholesterol, *LDL-P* low density lipoprotein particle

Comparing the type 1 cohort to the combined type 2 cohorts, the type 1 diabetes patient cohort was about 20 years younger and had numerically lower body mass index (BMI), higher HDL-C, and lower serum TGs (Table 1). The type 1 diabetes cohort also differed from the type 2 diabetes cohort at baseline in having numerically lower mean LFC, higher large and medium HDL, lower small HDL, higher large and lower small LDL, and lower VLDL subclass concentrations, and higher adiponectin levels (Table 1). Baseline values for some of the lipoprotein subclass and other lipid parameters were positively correlated with the baseline LFC (Table 2). The correlations reaching statistical significance were with large VLDL (all cohorts), Apo A2 (type 1 cohort), and Apo C3 (type 1 and type 2 basal switch cohorts).

**Table 2:** Correlations of baseline LFC with baseline lipid parameters

	Type 1 diabetes	T2 insulin naïve	T2 basal switch
	N = 249	N = 178	N = 225
Lipoprotein concentration			
HDL-P, μmol/L			
Total HDL	0.217	0.054	0.242
Large HDL	−0.094	−0.193	−0.216
Medium HDL	0.096	0.232	0.123
Small HDL	0.177	0.139	0.262*
LDL-P, nmol/L			
Total LDL	0.228	0.190	0.206
IDL	0.205	0.197	0.076
Large LDL	−0.107	−0.184	−0.096
Small LDL	0.216	0.243	0.253*
VLDL-P, nmol/L			
Total VLDL	0.100	0.113	0.116
Large VLDL	0.294*	0.398*	0.474*
Medium VLDL	0.160	0.087	0.119
Small VLDL	−0.024	0.048	−0.002
Lipoprotein size			
HDL size, nm	−0.158	−0.183	−0.207
LDL size, nm	−0.187	−0.220	−0.183
VLDL size, nm	0.214	0.415*	0.460*
Apolipoproteins			
Apo A1, mg/dL	0.138	0.133	0.118
Apo A2, mg/dL	0.247*	0.200	0.125
Apo B100, mg/dL	0.216	0.194	0.181
Apo C3, mg/dL	0.263*	0.225	0.298*
Other parameters			
Adiponectin, ng/mL	−0.154	−0.251	−0.196
CETP, pmol/mL/min	0.009	−0.048	−0.031
CETP, μg/mL	−0.160	0.050	−0.004
Serum CEC, %	0.290*	−0.043	0.008
Free fatty acid, mEq/L	0.061	0.234	0.222

Data are Spearman r correlation coefficients

*CEC* cholesterol efflux capacity, *CETP* cholesterol ester transfer protein, *HDL-P* high density lipoprotein particle, *LDL-P* low density lipoprotein particle, *T2* type 2 diabetes, *VLDL-P* very low density lipoprotein particle

* p < 0.001 for correlation

### Type 1 diabetes—effects of glargine and BIL

**Fig. 1:**
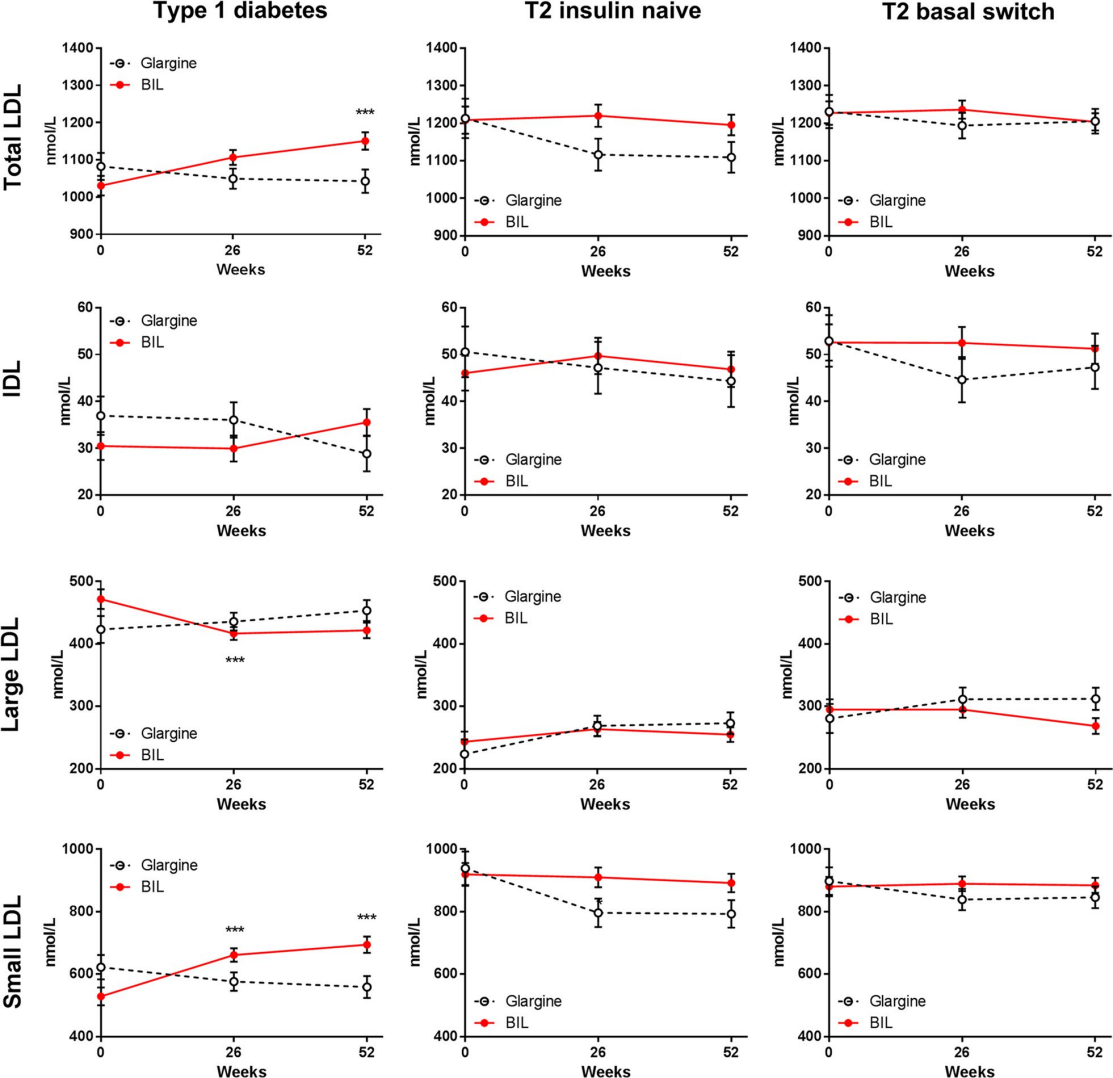
LDL subclass and IDL particle concentratons by treatment in three patient cohorts. Data are LS mean ± SE. p values are given for between-treatment differences where p < 0.001; ***p < 0.001 for change from baseline

**Fig. 2:**
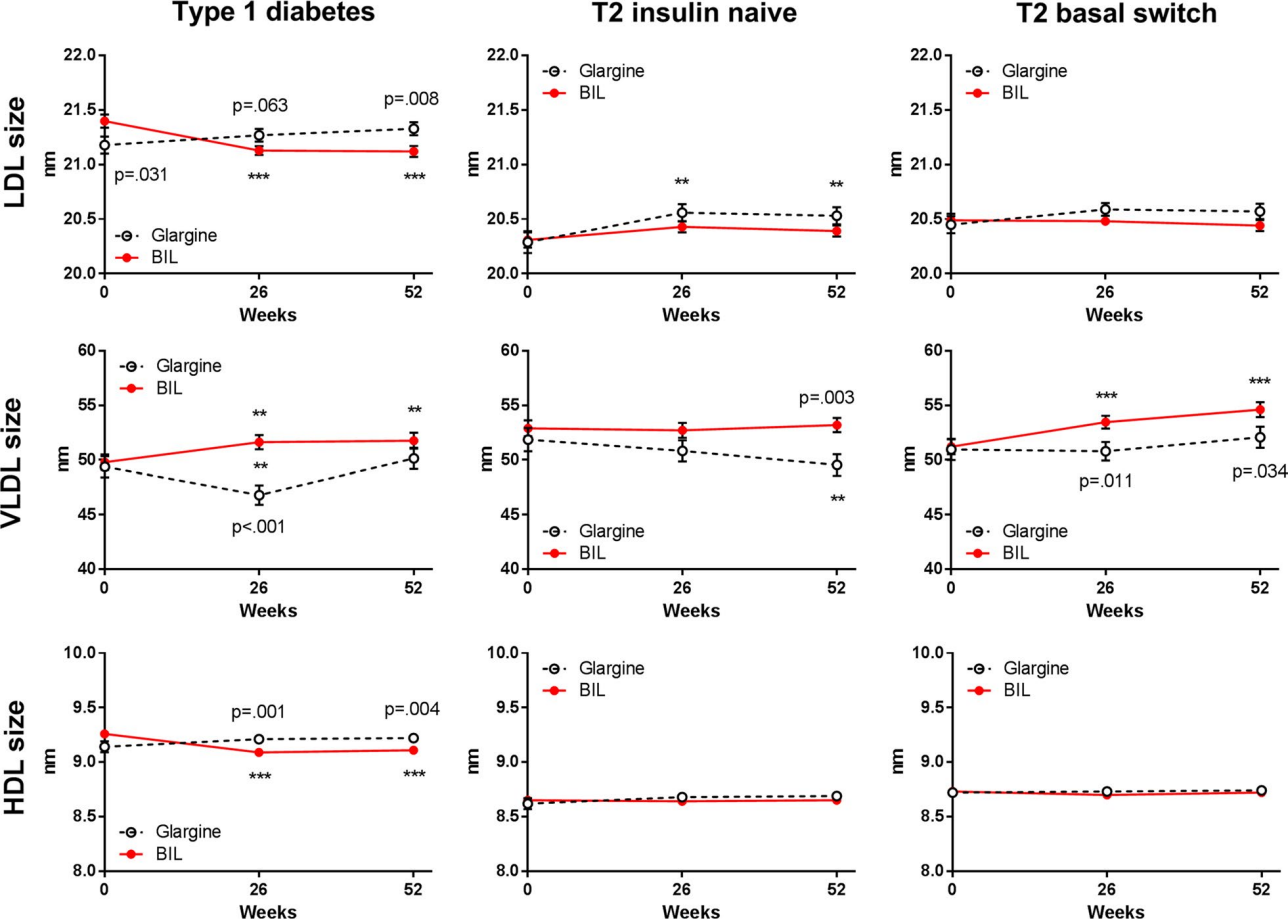
HDL, LDL, and VLDL particle size changes by treatment in three patient cohorts. Data are LS mean ± SE. p values are given for between-treatment differences where p < 0.001; ***p < 0.001 for change from baseline

Patients treated with glargine had no change from baseline in LDL particle concentration at 52 weeks, either in total LDL or in the large or small LDL subclasses (Fig. 1; Additional file 1: Table S1). In contrast, patients treated with BIL had a significant increase from baseline in total LDL at 52 weeks (Fig. 1; Additional file 1: Table S1). Among the LDL subclasses, large LDL concentrations were not significantly different between treatments at 26 or 52 weeks, although concentrations decreased significantly from baseline with BIL treatment at 26 weeks. However, small LDL concentrations showed significant increases from baseline with BIL treatment at both 26 and 52 weeks (Fig. 1; Additional file 1: Table S1). LDL particle size did not change with glargine treatment, but decreased significantly with BIL treatment from baseline to 26 and 52 weeks (Fig. 2; Additional file 1: Table S1).

**Fig. 3:**
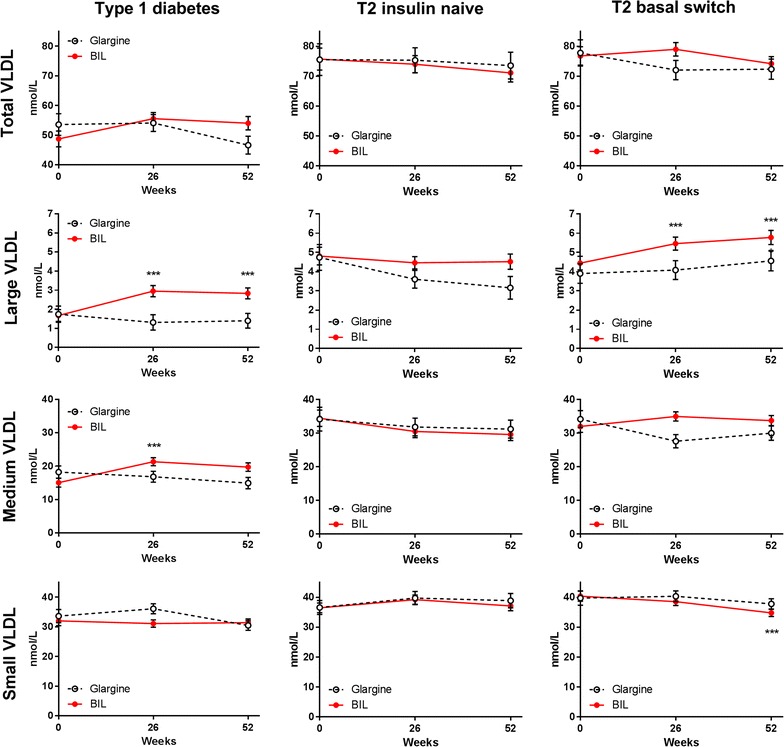
VLDL and VLDL subclass particle concentrations by treatment in three patient cohorts. Data are LS mean ± SE. p values are given for between-treatment differences where p < 0.001; ***p < 0.001 for change from baseline

**Fig. 4:**
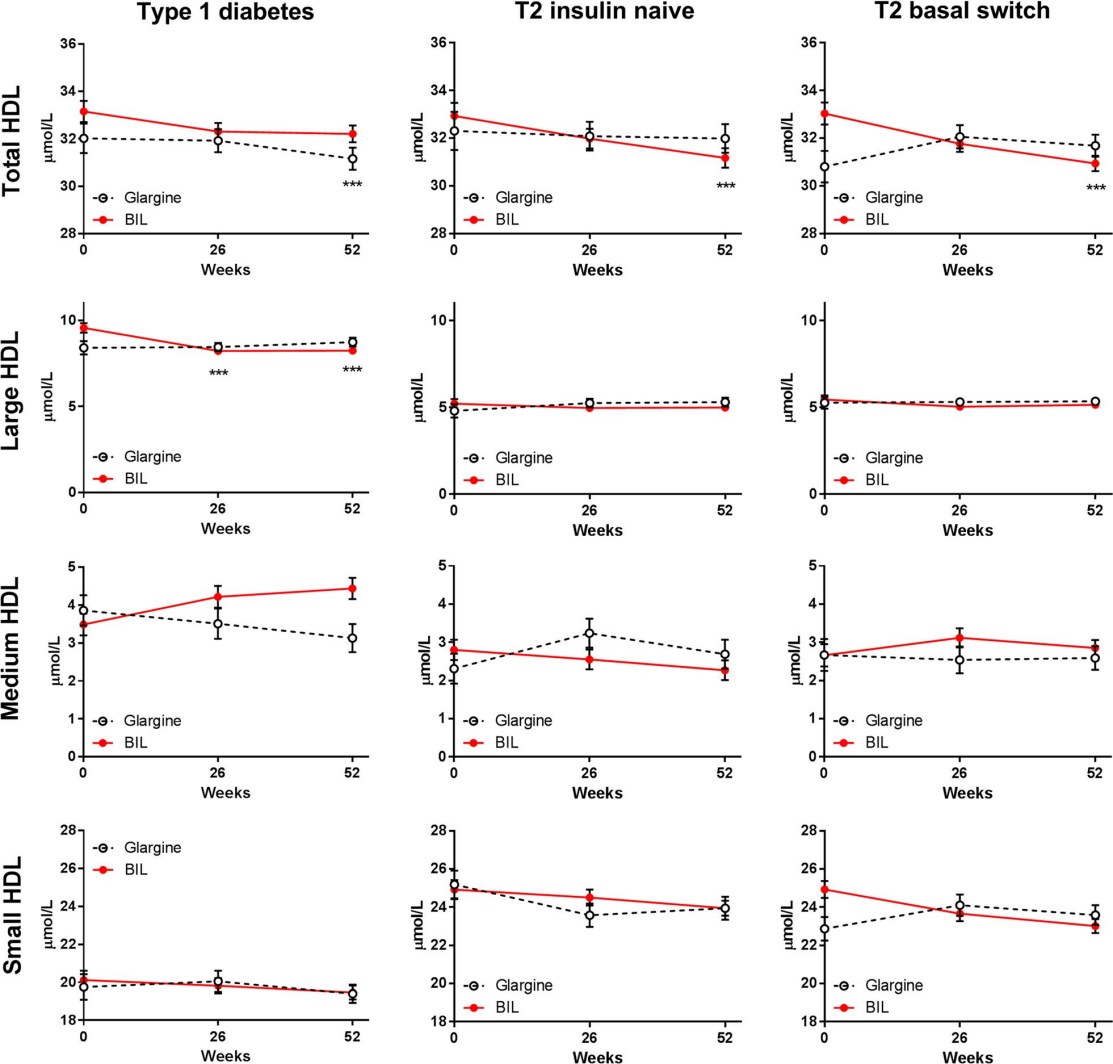
HDL and HDL subclass particle concentrations by treatment in three patient cohorts. Data are LS mean ± SE. p values are given for between-treatment differences where p < 0.001; ***p < 0.001 for change from baseline

Patients treated with glargine had no change in VLDL particle concentration over time, either in total VLDL or in the large, medium, or small VLDL subclasses (Fig. 3; Additional file 1: Table S1). Patients treated with BIL had significant increases from baseline in large and medium VLDL subclasses (Fig. 3; Additional file 1: Table S1). Total HDL particle concentration was not significantly different with BIL vs. glargine treatment, but large HDL decreased with BIL (Fig. 4; Additional file 1: Table S1).

Patients treated with glargine had no change in mean LFC, while patients treated with BIL had a mean increase in LFC [LSM difference at 52 weeks: 2.20% (1.26–3.13%); p < 0.001] (Additional file 1: Table S1) [8]. Positive correlations with changes in LFC were found for changes in medium HDL, large VLDL, and VLDL size, but only large VLDL in the glargine group correlated significantly (Table 3).

**Table 3:** Correlations of change in LFC with change in NMR parameters at 26 Weeks of treatment

	Type 1 diabetes		T2 insulin naïve		T2 basal switch	
	Glargine	BIL	Glargine	BIL	Glargine	BIL
	n = 86	n = 163	n = 59	n = 119	n = 74	n = 151
Lipoprotein concentration^a^						
HDL-P, μmol/L						
Total HDL	0.142	0.067	−0.051	0.158	−0.028	−0.072
Large HDL	−0.007	−0.017	−0.163	0.065	−0.114	−0.208
Medium HDL	0.132	0.214	0.050	−0.057	0.184	0.036
Small HDL	0.029	−0.048	−0.001	0.127	−0.101	−0.003
LDL-P, nmol/L						
Total LDL	0.088	−0.004	0.236	0.259	−0.106	0.201
IDL	0.163	0.099	0.025	0.389	0.180	0.004
Large LDL	−0.131	0.110	−0.033	−0.097	−0.214	−0.068
Small LDL	0.094	−0.037	0.305	0.235	−0.059	0.275
VLDL-P, nmol/L						
Total VLDL	0.171	0.093	0.045	0.318	−0.370	0.003
Large VLDL	0.480*	0.297	0.222	0.172	0.229	0.196
Medium VLDL	0.089	0.010	−0.024	0.186	−0.375	0.018
Small VLDL	0.144	0.079	0.081	0.332	−0.352	−0.057
Lipoprotein size^a^						
HDL size, nm	0.064	−0.057	−0.239	−0.180	0.047	−0.136
LDL size, nm	−0.173	0.164	−0.016	−0.220	−0.014	−0.216
VLDL size, nm	0.119	0.253	0.365	0.026	0.579*	0.189
Apolipoproteins^a^						
Apo A1, mg/dL	0.166	0.119	−0.014	0.131	0.017	−0.114
Apo A2, mg/dL	−0.011	0.180	0.184	0.335	0.147	0.124
Apo B100, mg/dL	0.077	0.042	0.161	0.510*	−0.033	0.227
Apo C3, mg/dL	0.403	0.138	0.015	0.393	−0.051	0.127
Other parameters^a^						
Adiponectin, ng/mL	−0.027	0.217	0.092	0.044	0.154	−0.137
CETP, pmol/mL/min	−0.106	−0.020	0.084	−0.038	−0.228	0.167
CETP, μg/mL	−0.361	−0.082	−0.152	0.041	−0.408	−0.137
Serum CEC, %	0.090	0.088	−0.159	0.118	−0.032	0.008
Free fatty acid, mEq/L	−0.016	0.097	0.322	0.162	0.077	0.147

*CEC* cholesterol efflux capacity, *CETP* cholesterol ester transfer protein, *HDL-P* high density lipoprotein particle, *LDL-P* low density lipoprotein particle, *T2* type 2 diabetes, *VLDL-P* very low density lipoprotein particle

* p < 0.001 for correlation

^a^Data are Spearman r correlation coefficients

**Fig. 5:**
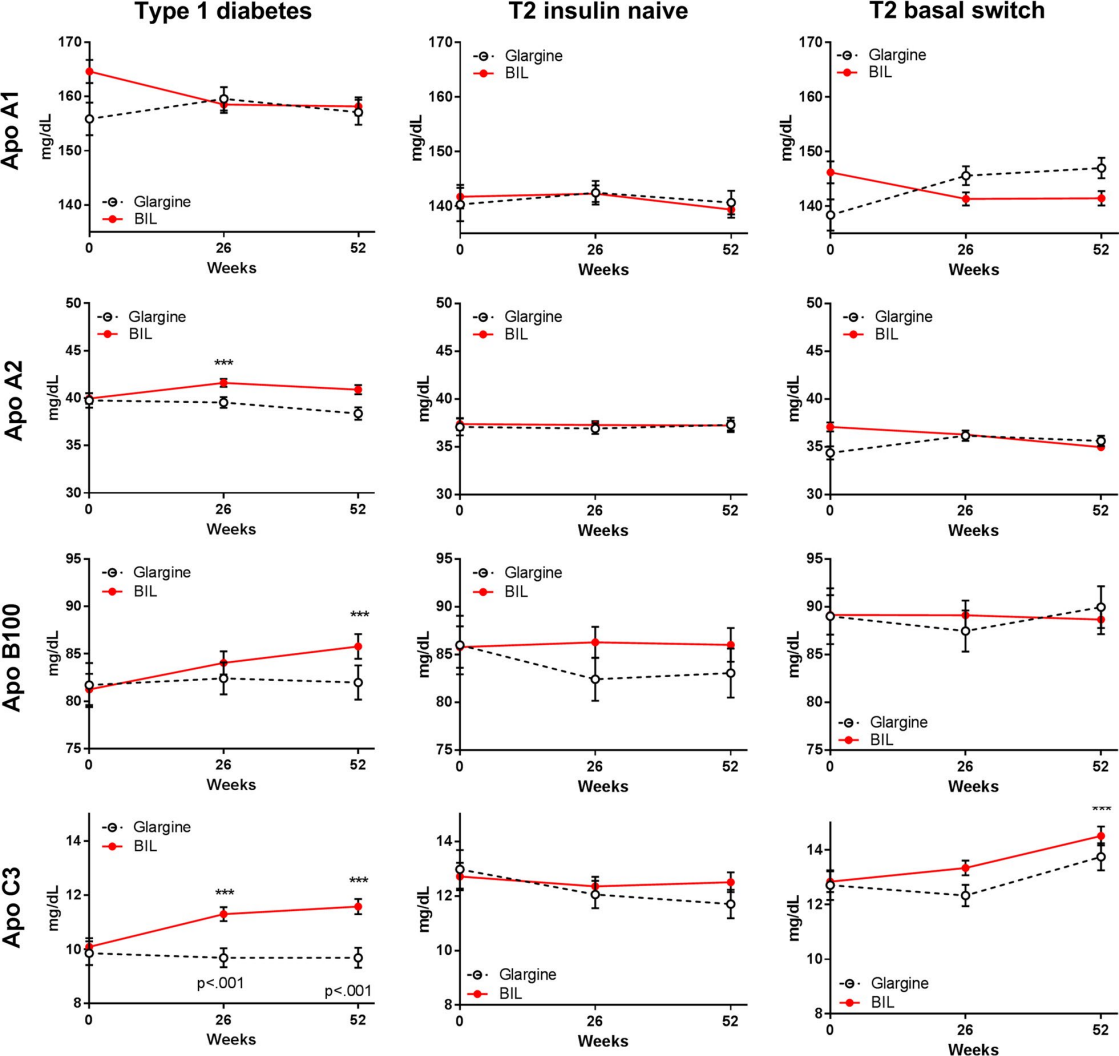
Apolipoprotein concentrations by treatment in three patient cohorts. Data are LS mean ± SE. p values are given for between-treatment differences where p < 0.001; ***p < 0.001 for change from baseline

**Fig. 6:**
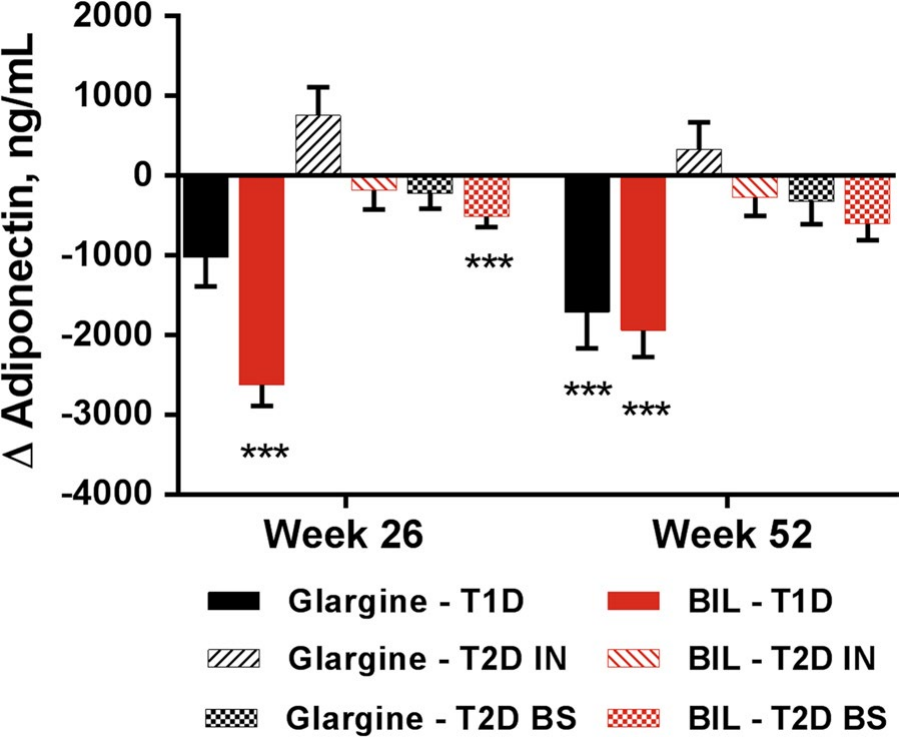
Adiponectin change from baseline to 26 and 52 weeks of treatment with glargine or BIL. Data are LS mean ± SE; all between treatment differences had p ≥ 0.001. ***p < 0.001 for change from baseline. *T1D* type 1 diabetes, *T2D* type 2 diabetes, *BS* basal switch cohort, *IN* insulin naïve cohort

Patients treated with glargine had no significant changes in the apolipoproteins measured (Fig. 5; Additional file 1: Table S1). Patients treated with BIL had significant increases from baseline in Apo A2, Apo B100, and Apo C3, with significant treatment differences at 26 and 52 weeks for Apo C3 (Fig. 5; Additional file 1: Table S1). There were no significant changes in CETP or CEC. Adiponectin concentrations decreased significantly from baseline to 52 weeks with both glargine and BIL treatment, with no significant difference between treatments (Fig. 6; Additional file 1: Table S1).

### Type 2 diabetes—effects of glargine and BIL

The two cohorts of type 2 diabetes patients were similar in baseline characteristics, with the exception that LFC was numerically higher in the insulin naïve cohort (Table 1). The insulin naïve cohort had a numerically higher concentration of large VLDL compared to the cohort previously taking insulin (basal switch) (Table 1).

### Insulin naïve cohort

Total LDL concentrations decreased from baseline with glargine treatment; the change was largely in the small LDL subclass (Fig. 1; Additional file 1: Table S1). Large VLDL decreased from baseline with glargine treatment and was significantly lower compared to the BIL group at 52 weeks (Fig. 3; Additional file 1: Table S1). Compared to baseline, VLDL size decreased while LDL size increased with glargine treatment (Fig. 2; Additional file 1: Table S1). Total HDL concentrations were unchanged with glargine treatment but decreased from baseline with BIL treatment (Fig. 4; Additional file 1: Table S1).

Mean LFC decreased from baseline in patients treated with glargine, while there was no significant change with BIL treatment [LSM difference at 52 weeks: 2.57% (0.94–4.21%); p = 0.002] (Additional file 1: Table S1) [8]. Stronger correlations were found for changes in lipoprotein parameters with changes in LFC in patients taking BIL in this population (Table 3). The strongest correlations were with total LDL, IDL, small LDL, total VLDL, small VLDL, Apo A2, Apo B100, and Apo C3 [all positive; five had correlation coefficients (r) > 0.3].

There were no significant changes from baseline or treatment differences in any measured apolipoproteins in either treatment group at 26 or 52 weeks (Fig. 5; Additional file 1: Table S1). FFAs decreased significantly from baseline in both treatment groups, but this decrease was significantly greater with glargine vs. BIL (Additional file 1: Table S1). There were no significant changes in CETP or CEC. Adiponectin concentrations increased with glargine and decreased with BIL, but these changes were not significant (Fig. 6).

### Basal switch cohort

Patients previously on insulin that were randomized to glargine did not have any significant changes from baseline to 52 weeks in total LDL or any LDL subclass during treatment (Fig. 1; Additional file 1: Table S1), or in total VLDL or any VLDL subclass (Fig. 4). Patients assigned to BIL had mean increases from baseline in the large VLDL subclass which were significantly different from the glargine group at 26 weeks (Fig. 3; Additional file 1: Table S1). The glargine group had no changes from baseline in concentrations of HDL or HDL subclass particles. The BIL group had a mean decrease from baseline in total HDL and small HDL particles, but there were no significant differences between treatments at any endpoint and there was a difference at baseline (Fig. 4; Additional file 1: Table S1).

Mean LFC did not change in the glargine group, but increased from baseline in the BIL group [LSM difference at 52 weeks: 5.27% (3.43–7.11%); p < 0.001] (Additional file 1: Table S1) [8]. Some correlations were found for changes in lipoprotein parameters with changes in LFC in patients taking BIL (Table 3). The highest correlations were with small LDL, large VLDL, and Apo B100 (all positive), but none of r values were >0.3.

Patients treated with glargine had no significant change from baseline in any measured apolipoproteins. The BIL group had a significant increase from baseline in Apo C3 at 52 weeks (Fig. 5; Additional file 1: Table S1). There were no significant changes in CETP or CEC. Adiponectin concentrations declined with both BIL and glargine treatment, but the change from baseline was statistically significant only with BIL at 26 weeks (Fig. 6; Additional file 1: Table S1).

## Discussion

The effects of currently available insulins on lipid profiles have been summarized by Chaudhuri [2]. Data from the Phase 3 clinical program for BIL—compared to insulin glargine or NPH—have also recently been reported [8]. However, the effects of insulin therapies on NMR-determined lipoprotein subclasses is limited to selected cohorts of patients with type 1 diabetes [19–24] and very small studies in type 2 diabetes [25–29]. What follows is a discussion of our findings in the context of the available literature.

### Lipoprotein subclasses in type 1 diabetes

Glargine treatment had little effect on LDL particles; BIL treatment led to increases from baseline in total LDL, small LDL, and Apo B100, but a decrease in large LDL. Glargine did not affect VLDL particles; BIL treatment led to increases from baseline in medium and large VLDL (similar in diameter range to the VLDL1 subfraction [30]), and an increase in Apo C3. Medium HDL was (nominally) lower with glargine, but increased with BIL treatment. These changes are consistent with the increase in serum TGs noted with BIL treatment in the Phase 2/3 clinical program [8].

In a cross-sectional study, patients with type 1 diabetes compared to healthy controls had lower medium VLDL, lower small HDL, and larger HDL size, with higher concentrations of large HDL [21]. Whether these differences were due to insulin treatment is not known. The effects of intensive insulin therapy on NMR lipoproteins were evaluated during the Diabetes Complications and Control Trial (DCCT) [24]. The results showed that intensive insulin therapy was associated with larger LDL diameter and lower levels of small LDL and small HDL.

The effects of these differences on coronary artery disease (CAD) risk were studied by Erbey et al. [20] in the Pittsburgh Epidemiology Study of Diabetes Complications (EDC). In a large cohort of patients with type 1 diabetes (n = 337), higher concentrations of small, dense LDL were associated with higher cholesterol, TGs, total LDL and lower HDL-C, and an increased risk of CAD. Soedamah et al. [22] also studied subjects with type 1 diabetes from the EDC cohort in a nested case (CAD, n = 59) control study (non-CAD, n = 59). In univariate analyses, lipid mass and particle concentrations of all three VLDL subclasses, and small and medium LDL were higher in cases than controls, while large HDL concentration was lower. Medium HDL was higher in patients with CAD and in the multivariate model was associated with CAD. Lyons et al. [23] evaluated lipoprotein subclasses and relationships with carotid intima media thickness (CIMT) in DCCT/EDIC participants. In analyses that adjusted for multiple variables, LDL particle concentrations were associated with internal CIMT in both sexes; LDL-C and Apo B were also associated with CIMT.

In our study, patients with type 1 diabetes treated with glargine did not have significant changes in any lipoprotein subclasses, presumably because the study treatment was similar to the patients’ pre-study treatment. In patients treated with BIL, lipoprotein changes were suggestive of an increased risk for CVD (although the magnitude of the change in CVD risk is uncertain). There were too few cardiovascular events in the BIL program to make assessments of any relationships with lipoprotein subclasses [31].

### Lipoprotein subclasses in type 2 diabetes

In the type 2 diabetes insulin naïve cohort, glargine use was associated with nominal decreases from baseline in total and small LDL and large VLDL; these changes were not seen with BIL treatment. In contrast, patients with type 2 diabetes previously on insulin that were randomized to glargine (basal switch cohort) did not have these changes, suggesting effects of prior insulin treatment. Randomization to BIL treatment was associated with increased large VLDL in the basal switch cohort, which was concordant with the observed changes in TGs [8].

Several small, short term studies evaluated insulin effects on lipid subfractions in type 2 diabetes, which showed that insulin treatment was associated with lower concentrations of TG, VLDL, small LDL, and small HDL, as well as increased activity of adipose tissue lipoprotein lipase (LPL) and CETP, and decreased activity of hepatic lipase [26–29, 32]. These changes are consistent with the known effects of insulin in lowering circulating TG [2], and the subsequent effects of TG levels on lipoproteins [33].

Although the effects of conventional insulin on serum TGs have been previously reported [2], data for the effects of insulin on VLDL particles are limited. In the overall BIL program, TGs increased from baseline when patients who were previous taking insulin were switched to BIL, but not when insulin naïve patients were treated with BIL [8]. In the current study, changes in large VLDL most closely paralleled these observations (as did, to a lesser extent, VLDL size), suggesting that the observed differences between glargine and BIL hinged on changes in the large VLDL subclass. Glargine has a greater peripheral insulin effect than BIL and thus would be expected to increase peripheral lipogenesis, while reducing lipolysis [4]. This may be mediated via increased fatty acid uptake subsequent to increased LPL activity [25]. BIL would have less effect on peripheral lipogenesis, and would allow increased peripheral lipolysis compared to glargine, which would likely result in increased FFA levels and hepatic VLDL production. The studies noted above did not report surrogate imaging markers of CVD to assess effects of lipid subfractions on CVD risk. In the BIL Phase 3 program, there were too few clinical CV events in patients with type 2 diabetes to assess any relationship of these lipid fractions with clinical disease [31].

### Adiponectin

There is extensive literature describing adiponectin as an adipokine that is associated with insulin sensitivity and vascular wall anti-inflammatory activity in type 2 diabetes, and showing that lower levels of adiponectin are associated with insulin resistance in type 2 diabetes [34–38]. However, there are only limited data in patients with type 1 diabetes. In the current study, the type 1 diabetes cohort had much higher mean adiponectin levels than the type 2 diabetes cohorts at baseline. Other investigators have reported that adiponectin levels are higher in patients with type 1 diabetes compared to non-diabetes controls [39–43]. The observations from the current study that adiponectin levels decreased from baseline with both BIL and glargine treatment are also concordant with literature showing that adiponectin levels decline with intensification of insulin treatment [42–44]. In summary, the current data confirm that patients with type 1 diabetes may have high adiponectin levels, but whether lower levels of adiponectin are associated with insulin resistance or clinical cardiovascular disease cannot be confirmed.

### Associations with liver fat content

Increased LFC has been associated with increased risk for CVD [45–47]. Whether this is the result of “common soil” with other markers of insulin resistance is unclear. However, the data from the current study characterizes some of the associations between LFC and the NMR profile of lipoproteins and apolipoproteins.

Liver fat content was assessed at baseline and after insulin treatment in these diabetes cohorts [12]. In correlations of baseline LFC with baseline values of lipoproteins and other soluble biomarkers, we found that large VLDL, VLDL size, Apo C3, and small LDL had the strongest and most consistent positive correlations with LFC, with r values of 0.216‒0.460 across the diabetes cohorts. There were also positive correlations with small HDL and Apo B100, and a negative correlation with adiponectin. These parameters are all associated with features of insulin resistance.

These observations support the concept that LFC is a function of insulin resistance not only in type 2 diabetes, but also in type 1 diabetes. The negative baseline correlations of LFC with adiponectin, an insulin-sensitizing and anti-steatotic adipokine, are consistent with previous observations that lower levels of adiponectin are associated with higher LFC [48]. Of note, even in the type 1 diabetes cohort that had a much higher baseline adiponectin compared to the type 2 diabetes cohorts, the negative baseline relationship of adiponectin to LFC was present. These data suggest that even with lower LFC in type 1 diabetes than in type 2 diabetes, some of the same mechanisms of insulin resistance may be operative.

Because glargine treatment reduced LFC in insulin naïve patients, and patients randomized to BIL who had previously been on insulin had increases in LFC, we examined the correlations between changes in the various lipid parameters, change in LFC, and treatment (Table 3). In patients with type 1 diabetes treated with glargine, there was very little absolute change in LFC and few major correlations of LFC change with lipoprotein changes, except for a direct association with large VLDL. In insulin naïve patients with type 2 diabetes treated with glargine, there was a decrease in LFC that correlated positively with changes in VLDL measures; this effect was even stronger in patients with type 2 diabetes previously treated with insulin, where there was only a small decrease in LFC with glargine.

These observations suggest that no single lipoprotein subclass, apolipoprotein or even adiponectin is a major driver of change in LFC in these insulin-treated patients with diabetes, although large VLDL, VLDL size, Apo B100, and Apo C3 may be related in some scenarios.

### Strengths and limitations

The strengths of this study include the carefully characterized subgroup of patients from a large Phase 3 program in which three cohorts of patients with different diabetes types were studied [5]. There was uniform collection of serial lipid profiles and LFC, measures of glycemic efficacy, insulin dosing and adverse event profiles. The MRI data for LFC and the lipid and lipoprotein measures were all collected in standardized fashion across the substudy cohort. Background glucose- and lipid-lowering medications were stable before randomization and during the study and did not differ between treatment groups.

Limitations of this study include: (1) The substudy cohort was not representative of the whole Phase 3 cohort in terms of randomization and outcomes, as the ability to perform NMR/MRI was limited to certain study sites. (2) The study cohort is too small to do extensive multivariable analyses of whether such things as sex, glycemic control, background glucose-lowering medications, lipid-lowering medications, or changes in body weight affected lipids and lipoproteins or LFC. (3) Although elevated TG (and VLDL TG) and nonalcoholic fatty liver have been associated with hepatic insulin resistance [49, 50], we did not obtain insulin levels needed to calculate insulin resistance because of limited ability to interpret plasma insulin concentrations in insulin-treated patients. (4) Although we did multiplicity adjustments, the results for individual lipoprotein parameters should be interpreted with caution. (5) Although in patients treated with BIL lipoprotein particle and apoprotein changes were suggestive of increased CVD risk (although the magnitude of the change in CVD risk is uncertain), analyses related to clinical CVD risk or events were not feasible in this study. (6) Individual studies did not capture all of the details of all potential variables that might affect lipid values, including menopausal status and hormone replacement therapy.

## Conclusions

In conclusion, the data from the lipid substudy of the BIL Phase 3 program in insulin-treated cohorts of both type 1 and type 2 diabetes, with multiple measures of soluble biomarkers related to lipid/lipoprotein concentrations, provide a unique and extensive database on the effects of insulin glargine and an investigational hepato-preferential insulin (BIL). These data, particularly the demonstration of differences between insulin naïve and previously insulin-treated patients with type 2 diabetes, may be helpful toward understanding the effects of future insulin treatments [51] that may exhibit varying peripheral and hepatic effects on lipid metabolism and possibly CVD risk.

## Additional file

**Additional file 1: Table S1.** Change in LFC, NMR lipoproteins, and other parameters from baseline to 26 and 52 weeks of treatment.
