# Supplementary material for: The effects of basal insulin peglispro vs. insulin glargine on lipoprotein particles by NMR and liver fat content by MRI in patients with diabetes
Source: Cardiovasc Diabetol. 2017 Jun 6;16:73. doi: 10.1186/s12933-017-0555-1 (PMC5461740; doi:10.1186/s12933-017-0555-1)
Supplement: Supplementary file 1 — Additional file 1: Table S1. Change in LFC, NMR lipoproteins, and other parameters from baseline to 26 and 52 weeks of treatment. [file 12933_2017_555_MOESM1_ESM.docx]

# APPENDIX

# Table S1. Change in LFC, NMR lipoproteins, and other parameters from baseline to 26 and 52 weeks of Treatment

| Patient cohort | Change from Baseline to Week 26 | | | | | Change from Baseline to Week 52 | | | | | | | |  |
| --- | --- | --- | --- | --- | --- | --- | --- | --- | --- | --- | --- | --- | --- | --- |
|  | **n**  **GL/BIL** | **Glargine** | **BIL** | | **p-value^†^** | **n**  **GL/BIL** | **Glargine** | | | **BIL** | | | **p-value^†^** |  |
| Liver fat content, % | | | | | | | | | | | | | | |
| T1D | 64/118 | -0.18 ± 0.32 | 2.23 ± 0.23* | | <0.001 | 57/107 | | 0.32 ± 0.38 | | 2.52 ± 0.28* | | | <0.001 |  |
| T2D insulin naive | 55/109 | -3.79 ± 0.59* | -0.78 ± 0.42 | | <0.001 | 47/94 | | -3.14 ± 0.67* | | -0.57 ± 0.48 | | | 0.002 |  |
| T2D basal switch | 52/108 | -1.09 ± 0.69 | 4.93 ± 0.48* | | <0.001 | 44/92 | | -0.56 ± 0.76 | | 4.71 ± 0.53* | | | <0.001 |  |
| Lipoprotein concentrations | | | | | | | | | | | | | | |
| Total HDL, μmol/L | | | | | | | | | | | | | | |
| T1D | 75/141 | -0.89 ± 0.49 | -0.50 ± 0.36 | 0.52 | | 73/130 | | -1.66 ± 0.47* | | -0.61 ± 0.35 | | | 0.79 |  |
| T2D insulin naive | 45/90 | -0.63 ± 0.60 | -0.75 ± 0.42 | 0.87 | | 44/94 | | -0.74 ± 0.60 | | -1.55 ± 0.41* | | | 0.27 |  |
| T2D basal switch | 68/138 | -0.16 ± 0.49 | -0.45 ± 0.34 | 0.62 | | 59/119 | | -0.53 ± 0.46 | | -1.27 ± 0.32* | | | 0.19 |  |
| Large HDL, μmol/L | | | | | | | | | | | | | | |
| T1D | 75/141 | -0.63 ± 0.24 | -0.86 ± 0.18* | 0.44 | | 73/130 | | -0.35 ± 0.26 | | -0.85 ± 0.20* | | | 0.13 |  |
| T2D insulin naive | 45/90 | 0.13 ± 0.25 | -0.16 ± 0.18 | 0.35 | | 44/94 | | 0.18 ± 0.26 | | -0.13 ± 0.17 | | | 0.32 |  |
| T2D basal switch | 68/138 | -0.09 ± 0.19 | -0.36 ± 0.13 | 0.25 | | 59/119 | | -0.05 ± 0.21 | | -0.25 ± 0.15 | | | 0.45 |  |
| Medium HDL, μmol/L | | | | | | | | | | | | | | |
| T1D | 75/141 | -0.09 ± 0.40 | -0.62 ± 0.29 | 0.15 | | 73/130 | | -0.47 ± 0.37 | | 0.84 ± 0.28 | | | 0.005 |  |
| T2D insulin naive | 45/90 | 0.60 ± 0.38 | -0.09 ± 0.26 | 0.13 | | 44/94 | | 0.05 ± 0.38 | | -0.37 ± 0.26 | | | 0.36 |  |
| T2D basal switch | 68/138 | -0.11 ± 0.35 | 0.47 ± 0.25 | 0.18 | | 59/119 | | -0.05 ± 0.31 | | 0.21 ± 0.21 | | | 0.49 |  |
| Small HDL, μmol/L | | | | | | | | | | | | | | |
| T1D | 75/141 | -0.08 ± 0.56 | -0.31 ± 0.41 | | 0.74 | 73/130 | | -0.73 ± 0.49 | | -0.67 ± 0.37 | | | 0.93 |  |
| T2D insulin naive | 45/90 | -1.39 ± 0.61 | -0.47 ± 0.42 | | 0.22 | 44/94 | | -1.01 ± 0.60 | | -1.01 ± 0.40 | | | >0.99 |  |
| T2D basal switch | 68/138 | -0.07 ± 0.56 | -0.51 ± 0.39 | | 0.53 | 59/119 | | -0.57 ± 0.52 | | -1.16 ± 0.37 | | | 0.36 |  |
| Total LDL, nmol/L | | | | | | | | | | | | | | |
| T1D | 75/141 | -8.76 ± 27.5 | 48.2 ± 20.0 | | 0.096 | 73/130 | | -15.6 ± 31.1 | | 92.3 ± 23.2* | | | 0.006 |  |
| T2D insulin naive | 45/90 | -95.6 ± 42.4 | 8.16 ± 29.5 | | 0.047 | 44/94 | | -103 ± 40.8 | | -16.5 ± 27.6 | | | 0.084 |  |
| T2D basal switch | 68/138 | -25.1 ± 34.3 | 17.3 ± 24.1 | | 0.31 | 59/119 | | -13.5 ± 32.9 | | -15.3 ± 23.1 | | | 0.97 |  |
| IDL, nmol/L | | | | | | | | | | | | | | |
| T1D | 75/141 | 3.50 ± 3.79 | -2.59 ± 2.75 | | 0.20 | 73/130 | | -3.68 ± 3.78 | | | 3.02 ± 2.83 | | 0.16 |  |
| T2D insulin naive | 45/90 | -0.59 ± 5.53 | 1.94 ± 3.87 | | 0.71 | 44/94 | | -3.41 ± 5.54 | | | -0.93 ± 3.77 | | 0.71 |  |
| T2D basal switch | 68/138 | -6.69 ± 4.84 | 1.14 ± 3.40 | | 0.19 | 59/119 | | -4.03 ± 4.62 | | | -0.09 ± 3.25 | | 0.49 |  |
| Large LDL, nmol/L | | | | | | | | | | | | | | |
| T1D | 75/141 | -19.0 ± 14.2 | -37.8 ± 10.3* | | 0.29 | 73/130 | -1.14 ± 16.8 | | | | -32.9 ± 12.5 | | 0.13 |  |
| T2D insulin naive | 45/90 | 26.4 ± 16.4 | 21.0 ± 11.5 | | 0.79 | 44/94 | 30.6 ± 17.3 | | | | 12.3 ± 11.8 | | 0.39 |  |
| T2D basal switch | 68/138 | 18.8 ± 18.9 | 2.20 ± 13.2 | | 0.47 | 59/119 | 19.6 ± 17.7 | | | | -23.9 ± 12.4 | | 0.046 |  |
| Small LDL, nmol/L | | | | | | | | | | | | | | |
| T1D | 75/141 | 5.13 ± 29.8 | 89.8 ± 21.6* | | 0.023 | 73/130 | -12.3 ± 35.2 | | | 123 ± 26.3* | | | 0.002 |  |
| T2D insulin naive | 45/90 | -125 ± 45.6 | -12.1 ± 31.5 | | 0.044 | 44/94 | -129 ± 43.9 | | | -29.8 ± 29.7 | | | 0.065 |  |
| T2D basal switch | 68/138 | -36.4 ± 33.9 | 14.2 ± 23.8 | | 0.22 | 59/119 | -29.4 ± 34.5 | | | 8.89 ± 24.3 | | | 0.37 |  |
| Total VLDL, nmol/L | | | | | | | | | | | | | | |
| T1D | 75/141 | 3.49 ± 2.87 | 4.93 ± 2.09 | | 0.69 | 73/130 | -3.98 ± 3.00 | | | 3.41 ± 2.24 | | | 0.051 |  |
| T2D insulin naive | 45/90 | 0.33 ± 4.16 | -1.04 ± 2.90 | | 0.79 | 44/94 | -1.46 ± 4.49 | | | -3.93 ± 3.04 | | | 0.65 |  |
| T2D basal switch | 68/138 | -4.09 ± 3.22 | 2.83 ± 2.26 | | 0.081 | 59/119 | -3.80 ± 3.39 | | | -1.94 ± 2.39 | | | 0.66 |  |
| Large VLDL, nmol/L | | | | | | | | | | | | | | |
| T1D | 75/141 | -0.30 ± 0.40 | 1.34 ± 0.29* | | 0.001 | 73/130 | -0.21 ± 0.39 | | | 1.23 ± 0.29* | | | 0.004 |  |
| T2D insulin naive | 45/90 | -1.09 ± 0.46 | -0.23 ± 0.32 | | 0.13 | 44/94 | -1.53 ± 0.59 | | | -0.17 ± 0.40 | | | 0.057 |  |
| T2D basal switch | 68/138 | -0.08 ± 0.49 | 1.31 ± 0.34* | | 0.021 | 59/119 | 0.40 ± 0.52 | | | 1.62 ± 0.37* | | | 0.059 |  |
| Medium VLDL, nmol/L | | | | | | | | | | | | | | |
| T1D | 75/141 | 0.90 ± 1.65 | 5.40 ± 1.20* | | 0.029 | 73/130 | -0.98 ± 1.70 | | | 3.81 ± 1.27 | | | 0.026 |  |
| T2D insulin naive | 45/90 | -1.39 ± 2.63 | -2.75 ± 1.83 | | 0.68 | 44/94 | -2.06 ± 2.68 | | | -3.63 ± 1.82 | | | 0.63 |  |
| T2D basal switch | 68/138 | -4.59 ± 1.98 | 2.79 ± 1.39 | | 0.003 | 59/119 | -2.20 ± 2.16 | | | 1.54 ± 1.52 | | | 0.16 |  |
| Small VLDL, nmol/L | | | | | | | | | | | | | | |
| T1D | 75/141 | 2.98 ± 1.70 | -2.00 ± 1.24 | | 0.019 | 73/130 | -2.65 ± 1.67 | | | -1.68 ± 1.25 | | | 0.64 |  |
| T2D insulin naive | 45/90 | 2.66 ± 2.21 | 2.11 ± 1.55 | | 0.84 | 44/94 | 1.80 ± 2.41 | | | 0.02 ± 1.64 | | | 0.54 |  |
| T2D basal switch | 68/138 | 0.54 ± 1.80 | -1.25 ± 1.26 | | 0.42 | 59/119 | -1.99 ± 1.73 | | | -5.03 ± 1.22* | | | 0.15 |  |
| Lipoprotein size | | | | | | | | | | | | | | |
| HDL size, nm | | | | | | | | | | | | | | |
| T1D | 75/141 | 0.01 ± 0.03 | -0.11 ± 0.02* | | 0.001 | 73/130 | 0.01 ± 0.03 | | | -0.09 ± 0.02* | | | 0.004 |  |
| T2D insulin naive | 45/90 | 0.04 ± 0.03 | 0.00 ± 0.02 | | 0.41 | 44/94 | 0.05 ± 0.03 | | | 0.01 ± 0.02 | | | 0.36 |  |
| T2D basal switch | 68/138 | 0.00 ± 0.03 | -0.03 ± 0.02 | | 0.29 | 59/119 | 0.01 ± 0.03 | | | -0.01 ± 0.02 | | | 0.66 |  |
| LDL size, nm | | | | | | | | | | | | | | |
| T1D | 75/141 | -0.04 ± 0.06 | -0.18 ± 0.04* | | 0.063 | 73/130 | 0.02 ± 0.06 | | | -0.19 ± 0.05* | | | 0.008 |  |
| T2D insulin naive | 45/90 | 0.25 ± 0.08 | 0.12 ± 0.05 | | 0.17 | 44/94 | 0.21 ± 0.08 | | | 0.08 ± 0.05 | | | 0.14 |  |
| T2D basal switch | 68/138 | 0.10 ± 0.06 | -0.01 ± 0.04 | | 0.18 | 59/119 | 0.08 ± 0.07 | | | -0.06 ± 0.05 | | | 0.12 |  |
| VLDL size, nm | | | | | | | | | | | | | | |
| T1D | 75/141 | -2.67 ± 0.89 | 2.15 ± 0.65 | | <0.001 | 73/130 | 0.67 ± 0.97 | | | 2.29 ± 0.73 | | | 0.18 |  |
| T2D insulin naive | 45/90 | -1.53 ± 0.98 | 0.35 ± 0.69 | | 0.12 | 44/94 | -2.82 ± 0.98 | | | 0.83 ± 0.66 | | | 0.003 |  |
| T2D basal switch | 68/138 | -0.18 ± 0.85 | 2.48 ± 0.59* | | 0.011 | 59/119 | 1.11 ± 0.97 | | | 3.63 ± 0.68* | | | 0.034 |  |
| Apolipoproteins | | | | | | | | | | | | | | |
| Apo A1, mg/dL | | | | | | | | | | | | | | |
| T1D | 77/146 | -1.37 ± 2.17 | -2.43 ± 1.56 | | 0.69 | 72/132 | -3.85 ± 2.30 | | | -2.79 ± 1.69 | | | 0.71 |  |
| T2D insulin naive | 48/94 | 1.16 ± 2.16 | 1.00 ± 1.53 | | 0.95 | 47/98 | -0.64 ± 2.17 | | | -1.94 ± 1.50 | | | 0.62 |  |
| T2D basal switch | 70/139 | 2.33 ± 1.73 | -1.94 ± 1.22 | | 0.047 | 60/123 | 3.72 ± 1.87 | | | -1.81 ± 1.30 | | | 0.017 |  |
| Apo A2, mg/dL | | | | | | | | | | | | | | |
| T1D | 78/146 | -0.32 ± 0.57 | 1.75 ± 0.42* | | 0.004 | 72/129 | -1.50 ± 0.66 | | | 1.03 ± 0.49 | | | 0.002 |  |
| T2D insulin naive | 48/94 | -0.29 ± 0.57 | 0.08 ± 0.41 | | 0.60 | 47/98 | 0.10 ± 0.76 | | | 0.03 ± 0.52 | | | 0.94 |  |
| T2D basal switch | 69/139 | 0.03 ± 0.54 | 0.15 ± 0.38 | | 0.86 | 59/121 | -0.52 ± 0.54 | | | -1.18 ± 0.37 | | | 0.32 |  |
| Apo B100, mg/dL | | | | | | | | | | | | | | |
| T1D | 72/139 | 1.09 ± 1.69 | 2.72 ± 1.21 | | 0.44 | 66/125 | 0.66 ± 1.81 | | | 4.45 ± 1.31* | | | 0.092 |  |
| T2D insulin naive | 46/86 | -3.24 ± 2.26 | 0.63 ± 1.62 | | 0.17 | 41/87 | -2.59 ± 2.58 | | | 0.34 ± 1.77 | | | 0.35 |  |
| T2D basal switch | 67/131 | -1.28 ± 2.15 | 0.38 ± 1.54 | | 0.53 | 58/117 | 1.21 ± 2.18 | | | -0.08 ± 1.54 | | | 0.63 |  |
| Apo C3, mg/dL | | | | | | | | | | | | | | |
| T1D | 78/146 | -0.27 ± 0.35 | 1.34 ± 0.26* | | <0.001 | 72/129 | -0.28 ± 0.37 | | | 1.62 ± 0.28* | | | <0.001 |  |
| T2D insulin naive | 48/94 | -0.43 ± 0.50 | -0.13 ± 0.35 | | 0.62 | 47/98 | -0.78 ± 0.52 | | | 0.01 ± 0.36 | | | 0.22 |  |
| T2D basal switch | 69/139 | -0.25 ± 0.39 | 0.76 ± 0.27 | | 0.035 | 59/121 | 1.17 ± 0.50 | | | 1.93 ± 0.35* | | | 0.22 |  |
| Other Parameters | | | | | | | | | | | | | | |
| Adiponectin, ng/mL | | | | | | | | | | | | | | |
| T1D | 75/140 | -1021 ± 368 | -2622 ± 268* | | 0.001 | 70/128 | -1708 ± 456* | | | -1939 ± 336* | | | 0.68 |  |
| T2D insulin naive | 47/92 | 757 ± 353 | -181 ± 244 | | 0.031 | 45/97 | 330 ± 341 | | | -272 ± 230 | | | 0.15 |  |
| T2D basal switch | 69/138 | -223 ± 188 | -512 ± 133* | | 0.21 | 60/121 | -321 ± 286 | | | -605 ± 201 | | | 0.42 |  |
| CETP, pmol/mL/min | | | | | | | | | | | | | | |
| T1D | 75/142 | -0.11 ± 0.44 | -0.06 ± 0.32 | | 0.92 | 73/127 | -0.20 ± 0.53 | | | -0.00 ± 0.40 | | | 0.77 |  |
| T2D insulin naive | 47/83 | 1.31 ± 0.53 | 1.65 ± 0.38* | | 0.60 | 45/88 | 1.97 ± 0.58* | | | 1.59 ± 0.41* | | | 0.60 |  |
| T2D basal switch | 68/137 | 0.95 ± 0.52 | 0.29 ± 0.36 | | 0.30 | 59/118 | 0.32 ± 0.54 | | | -0.07 ± 0.38 | | | 0.55 |  |
| CETP, μg/mL | | | | | | | | | | | | | | |
| T1D | 75/143 | -0.03 ± 0.04 | -0.10 ± 0.03* | | 0.094 | 73/129 | | | -0.06 ± 0.04 | | -0.08 ± 0.03 | | 0.69 |  |
| T2D insulin naive | 48/89 | 0.12 ± 0.03* | 0.11 ± 0.02* | | 0.87 | 45/96 | | | 0.13 ± 0.04* | | 0.12 ± 0.03* | | 0.77 |  |
| T2D basal switch | 69/138 | 0.06 ± 0.04 | -0.05 ± 0.03 | | 0.020 | 60/119 | | | 0.01 ± 0.04 | | -0.08 ± 0.03 | | 0.049 |  |
| Serum CEC, % | | | | | | | | | | | | | | |
| T1D | 75/144 | -0.30 ± 0.27 | -0.02 ± 0.19 | | 0.41 | 73/130 | | | -0.30 ± 0.22 | | -0.03 ± 0.16 | | 0.34 |  |
| T2D insulin naive | 46/91 | -0.07 ± 0.29 | -0.12 ± 0.20 | | 0.90 | 45/98 | | | 0.34 ± 0.31 | | -0.42 ± 0.21 | | 0.048 |  |
| T2D basal switch | 69/139 | 0.35 ± 0.27 | 0.26 ± 0.19 | | 0.78 | 60/122 | | | -0.22 ± 0.24 | | -0.45 ± 0.17 | | 0.43 |  |
| Free fatty acid, mEq/L | | | | | | | | | | | | | | |
| T1D | 72/139 | -0.02 ± 0.04 | 0.04 ± 0.03 | | 0.17 | 68/126 | | | -0.04 ± 0.04 | | | 0.07 ± 0.03 | 0.031 |  |
| T2D insulin naive | 47/89 | -0.16 ± 0.03* | -0.06 ± 0.02 | | 0.003 | 43/95 | | | -0.17 ± 0.03* | | | -0.08 ± 0.02* | 0.011 |  |
| T2D basal switch | 69/136 | -0.04 ± 0.04 | 0.03 ± 0.03 | | 0.12 | 58/122 | | | -0.03 ± 0.03 | | | -0.00 ± 0.02 | 0.38 |  |

^†^Unadjusted p-value for difference between treatments. *p<0.001 for change from baseline. Data are LS mean ± SE. BIL=basal insulin peglispro; GL=insulin glargine; T1D=type 1 diabetes;T2D=type 2 diabetes.
